# Fourth Intrnational Symposium on Metal Ions in Biology and Medicine

**DOI:** 10.1155/MBD.1995.124

**Published:** 1995


					FOURTH
INT RNATIONAL
SYMPOSIUM ON
M TAL IONS IN
BIOLOGY AND
M DICIN
Tarragona, Spain, May 19-22, 1996
The 4th International Symposium on Metal Ions in Biology and Medicine will be
held May 19-22, 1995, in Tarragona, Spain. The scientific program will include
plenary and keynote lectures, platform sessions, posters with discussion and
debates. This symposium will address the role of metal ions in areas of
biochemical, biological, and medical sciences. The symposium will be of
practical usefulness to those in basic and applied research, clinical scientists,
and researchers in allied medical fields.
For further information, please contact:
Prof. Dr. Jose L. Domingo
Chairman, 4th ISMIBM
Laboratory of Toxicology and
Biochemistry
School of Medicine
San Lorenzo 21,43201 Reus, Spain
Tel. 34-77-759380, Fax 34-77-759322
124

				

